# Raising the level: orangutans solve the floating peanut task without visual feedback

**DOI:** 10.1007/s10329-021-00952-4

**Published:** 2021-10-16

**Authors:** Carla Sebastián-Enesco, Nerea Amezcua-Valmala, Fernando Colmenares, Natacha Mendes, Josep Call

**Affiliations:** 1 Grupo UCM de Psicobiología social, evolutiva y comparada, Universidad Complutense de Madrid, Madrid, Spain; 2 Departamento de Investigación y Psicología en Educación, Facultad de Psicología, Universidad Complutense de Madrid, Madrid, Spain; 3 Departamento de Psicología. Facultad de Ciencias Biomédicas y Salud, Universidad Europea de Madrid, Madrid, Spain; 4 Departamento de Psicobiología y Metodología en Ciencias del Comportamiento, Facultad de Psicología, Universidad Complutense de Madrid, Madrid, Spain; 5 Max Planck School of Cognition, Max Planck Institute for Human Cognitive and Brain Sciences, Leipzig, Germany; 6 School of Psychology and Neuroscience, University of St Andrews, St. Andrews, UK

**Keywords:** Floating peanut task, Innovation, Insight, Visual feedback, Tool use, Orangutans

## Abstract

Chimpanzees and orangutans are able to generate innovative behaviors to solve complicated physical problems. For example, when presented with an out-of-reach peanut at the bottom of a vertical tube (floating peanut task—FPT), some of them spontaneously spit water into the tube until the peanut floats to the top. Yet, it is unclear whether this innovative solution results from repeating those actions that bring the peanut incrementally closer to the top or from anticipating the solution before acting. In the current study, we addressed this question by presenting three naïve orangutans with an opaque version of the FPT that prevented them from obtaining visual information about the effect of their actions on the position of the peanut. One of the subjects solved the opaque FPT in the very first trial: he collected water from the faucet and poured it into the opaque tube repeatedly until the hitherto non-visible peanut reached the top. This provides evidence for the first time that orangutans can potentially solve the FPT without relying on sensorimotor learning, but to some extent by mentally representing the problem.

**Supplementary Information:**

The online version contains supplementary material available at 10.1007/s10329-021-00952-4.

## Introduction

Great apes are proficient physical problem solvers that show high innovation rates in their tool use behavior compared to other nonhuman primates (Reader and Laland 2003). Innovation is usually referred to as the ability to invent new behaviors or use behaviors that are already part of one’s repertoire in a new way to solve unknown problems (Kummer and Goodall 1985). A novel solution may nevertheless result from a variety of cognitive processes, not necessarily mutually exclusive (Reader and Laland 2003), ranging from different types of sensorimotor mechanisms (e.g., trial-and-error learning, perceptual-motor feedback) to insight (Köhler 1926; Thorpe 1956) or mental combinations in Piagetian terms (i.e., mentally representing the problem and imagining both the solution and the sequence of means-action; Doré and Dumas 1987). Insight and mental combinations rely necessarily on some understanding of the causal relations involved in the problem that is activated before acting, whereas sensorimotor mechanisms mainly depend on the feedback generated by physically acting on the problem. Sensorimotor mechanisms can range from blind trial-and error to more sophisticated forms of operant conditioning and causal learning (which imply some understanding of the spatiotemporal relationships involved) (Skinner 1974; Thorndike 1911). The question of which cognitive mechanisms are responsible for innovative problem solving in great apes is still a matter of debate (Ramsey et al. 2007).

Mendes et al. (2007) designed a suitable task to explore innovation and pinpoint its underlying cognitive mechanisms in great apes: the floating peanut task (hereafter FPT). In this task, subjects are presented with a vertically oriented tube that contains an out-of-reach peanut at its bottom. The solution to this problem consists of pouring water into the tube so that the peanut floats upward until it reaches the tube opening. In the original study (Mendes et al. 2007), the peanut was already floating in a small amount of water inside a transparent tube (out of reach from the subject), and all five orangutans tested acquired the solution (i.e., collecting water from the faucets and spitting it into the tube) in the very first trial. Both the sudden appearance of the spitting behavior (which replaced previous ineffective actions) and the fact that it was only displayed to extract the peanut from the tube (compared to control conditions) suggest that orangutans engaged in insightful problem solving. Nevertheless, two features of the task precluded us from ruling out alternative explanations based on sensorimotor processes. First, the initial state of the problem (an object floating in water) provided information about its solution. That is, one can potentially solve the task by rearranging the key elements that were already present without engaging in genuine innovation. However, subsequent studies using a dry tube -thus removing the initial perceptual cues- debunked this interpretation (DeLong et al. 2020; Ebel et al. 2019b; Hanus et al. 2011). Some naïve chimpanzees and orangutans were able to solve the dry version of the FPT.

Second, and more important, the transparent tube allowed the individuals to visually monitor the effect of their spitting behavior on the position of the floating object. Therefore, subjects could have solved the transparent-tube task by some form of instrumental learning (i.e., repeating those actions that bring the reward incrementally closer), and not necessarily by means of mental combinations prior to action. As pointed out by Mendes et al. (2007) in the original study, a conclusive way to identify which cognitive mechanism may underlie the solution process in the FPT is presenting an opaque tube to naïve subjects that prevents them from seeing how their spitting responses positively affect the reward’s position. Recently, Ebel et al. (2019b) used the opaque version of the FPT in a series of experiments with great apes. None of the naïve subjects spontaneously solved the opaque task. However, success could be promoted by previously experiencing one of two sensorimotor scenarios (Ebel et al. 2019b). First, most of the subjects who succeeded in the dry transparent version of the FPT maintained the solution in the opaque version. Even the orangutans tested in the original study (Mendes et al. 2007) were able to transfer the solution, acquired 9 years earlier with the transparent quarter-filled water tube, to the opaque tube. Evidence for solution transference from simpler to more complex but analogous problems was also gathered from studies addressing other physical problems (e.g., Manrique et al. 2013). For instance, Völter and Call (2012) presented great apes a series of string-pulling tasks that varied in the visual access the subjects had to the different elements relevant for the solution process. Results show that when faced with complex problems, great apes only succeeded after a brief visual exposure to the effect of their actions in the solution process, but could maintain the solution afterwards in visually restricted versions of the task. Whether or not this is the result of the subjects identifying the common causal structure of both problems (simple and complex versions) needs further investigation. An alternative explanation is that the subjects repeated an action that worked in the past when facing the new task.

The second relevant finding of Ebel et al. (2019b) was the successful performance of a chimpanzee after observing an end-state demonstration in which the reward (peanut) was already floating atop the opaque tube within easy reach. Crucially, in this case, the solution was not acquired by perceptual-motor feedback. One possible (albeit speculative) explanation is that the individual tried to reproduce the observed result by generating new actions and anticipating their effect on the peanut’s position. In line with this interpretation, Tennie et al. (2010) found that chimpanzees could solve the transparent FPT apparently by attending to the end-state of the task produced by others, and spontaneously using their own means.

Altogether, these findings suggest that the great apes’ success in the FPT may not be only due to instrumental learning or entirely controlled by sensorimotor processes. Rather, it seems that great apes may use the sensorimotor information to generate a basic knowledge of the means-end relationships and physical properties of the task, which can be subsequently applied to similar problems. However, it is still unclear whether great apes can solve the FPT by mentally representing the effect of spitting water into an opaque tube without previously witnessing the effect. In the current study, we addressed this question by presenting three naïve orangutans with the opaque version of the FPT that prevented them from obtaining visual information about the task (same apparatus as in Ebel et al. 2019b). Unlike previous FPT studies, this study involved a fixed sequence of conditions of decreasing difficulty (i.e., from less to more visual information available regarding the tube’s content). This was done to ensure that success in more cognitively demanding FPT conditions was not due to transferring the solution from simpler versions. All subjects started with the opaque tube (opaque condition) and were subsequently presented with the transparent dry tube (dry condition). Success in the opaque condition would provide the first evidence that orangutans can solve the task without sensorimotor feedback, thus raising the possibility that individuals used mental re-combination to produce the solution. Subjects who failed in the opaque or dry tasks were then presented with a wet task, identical to the original transparent quarter-filled water tube (Mendes et al. 2007). If subjects solve the problem in the wet but not the dry condition, it would indicate that the presence of the floating peanut facilitated the solution. We ran a series of control conditions to assess whether subjects spat water indiscriminately into the tube regardless of the presence and/or position of the peanut. Crucially, we also included a control condition (dry-control) identical to the dry condition described above, to confirm that apes can transfer the successful sequence of actions to a more complex version of the task (e.g., Ebel et al. 2019b; Hanus et al. 2011; Mendes et al. 2007).

## Method

### Subjects

Three male orangutans housed at the Madrid Zoo (Spain) participated in the present study: Dahi (*Pongo pygmaeus*), and Ron and Tom (both *P. pygmaeus x P. abelii* hybrids), aged 10, 16, and 19 years, respectively. However, Tom (the flanged male) was withdrawn from the study due to his general lack of interest in the task. All subjects were housed in indoor-outdoor enclosures equipped with various enrichment structures (e.g., climbing frames, ropes, natural vegetation, water sources). Subjects were naïve to the task or any other experimental procedure. They participated voluntarily in the study and were not food- or water- deprived during the test. The experiment took place in their sleeping rooms where no tools were available.

### Materials

Two tubes were used in this study. The opaque tube (long = 26 cm; Ø = 5 cm) was closed at both ends and had a single hole (Ø ≈ 3 cm) near its top part (same as in Ebel et al. 2019b). Crucially, the size and position of the hole precluded the subjects from visually monitoring the peanut until it reached the hole. The transparent tube (long = 26 cm; Ø = 5 cm) was open at its top end (same as in Mendes et al. 2007). Depending on condition, either the opaque or the transparent tube was vertically attached to the bars of the sleeping room by metal bracelets at a distance of approx. 1.5 m from the water faucet. The faucets have been in the sleeping rooms since its construction, and subjects used them on a regular basis. There was no visual contact between the tested subject and other conspecifics.

### Procedure

#### Experimental conditions

Orangutans were first presented with the opaque condition, followed by the dry condition (see Supplementary file 1, Fig. 1 for a schematic drawing of experimental conditions). Each experimental condition consisted of four 25-min trials held in different days. The opaque trials began with the experimenter dropping the peanut into the dry opaque tube in full view of the subject. In this condition, the subjects were not able to use any visual cues to monitor the peanut inside the tube. In contrast, in the dry condition, the peanut was already inside the transparent dry tube and each dry trial began when the subject entered the room. Subjects who failed to retrieve the peanut in the opaque and dry conditions were tested in the wet-experimental condition that matched exactly the original FPT used in Mendes et al. 2007 study (i.e., a transparent tube filled with a quarter of water and the peanut floating inside but out of reach). The wet-experimental condition consisted of four 2-min trials administered on different days.

#### Control conditions

We administered four different control conditions: wet-control, top, table, and dry-control (see Supplementary file 1,Fig. 2 for a schematic drawing of control conditions). The wet-control condition was only presented to subjects who went through the wet-experimental condition and consisted of the very same water-filled transparent tube but without a peanut inside. This was done to assess whether the spitting behavior was exclusively due to the presence of water. The procedure and number of trials for the wet-control condition were the same as for the wet-experimental one. We counterbalanced the order of presentation of both wet conditions across subjects. The top, table, and dry-control conditions were administered at the end of the experiment to all subjects regardless of their performance in experimental conditions (same control conditions as in Mendes et al. 2007). In the top condition, a peanut was attached to the top of the transparent tube, and was thus within easy reach of the subject. This was done to rule out that subjects’ spitting behavior was triggered by the mere presence of the tube. In the table condition, a peanut rested on a table that was located outside the cage, and 31 cm away from the transparent tube and thus subjects could not reach it. We presented this condition to assess whether subjects’ spitting behavior was triggered by a frustration effect. The dry-control condition was identical to the dry condition except that each dry-control trial took 2 min. We included the dry-control condition to test whether subjects that only managed to get the peanut on the wet test condition would solve the task without the presence of water inside. Each of these control conditions involved four 2-min trials. Table, top and dry-control trials were grouped into four 3-trial blocks held in different days. Order of presentation within and between blocks was counterbalanced across subjects.

### Coding and analyses

All sessions were videotaped. For each trial, we coded whether and when the subjects solved the task (i.e., retrieved the peanut), as well as the latency to first spit into the tube and the time elapsed between successive water spits. Additionally, we scored the frequency of tube-directed actions that were ineffective for solving the task: hand/foot actions (pulling, lifting, banging) and mouth actions (biting, licking). A second coder coded 33% of the trials. Inter-observer reliability was excellent for all measurements (*Pearson*’s correlation coefficient: latencies to success, *r* = 1.00; latencies to spit, *r* = 1.00; number of spits, *r* = 1.00; tube-directed actions, *r* = .95).

## Results

The two orangutans succeeded in retrieving the peanut from the tube, albeit in different conditions. Dahi acquired the solution in the very first trial of the opaque condition and continued to succeed in all the following experimental sessions (four opaque and four dry sessions). Ron failed to solve the task during the opaque and dry conditions, and therefore was presented with the wet conditions. He managed to get the peanut in the first wet-experimental trial, and only needed one trial to succeed during the dry-control condition.

**Fig. 1:**
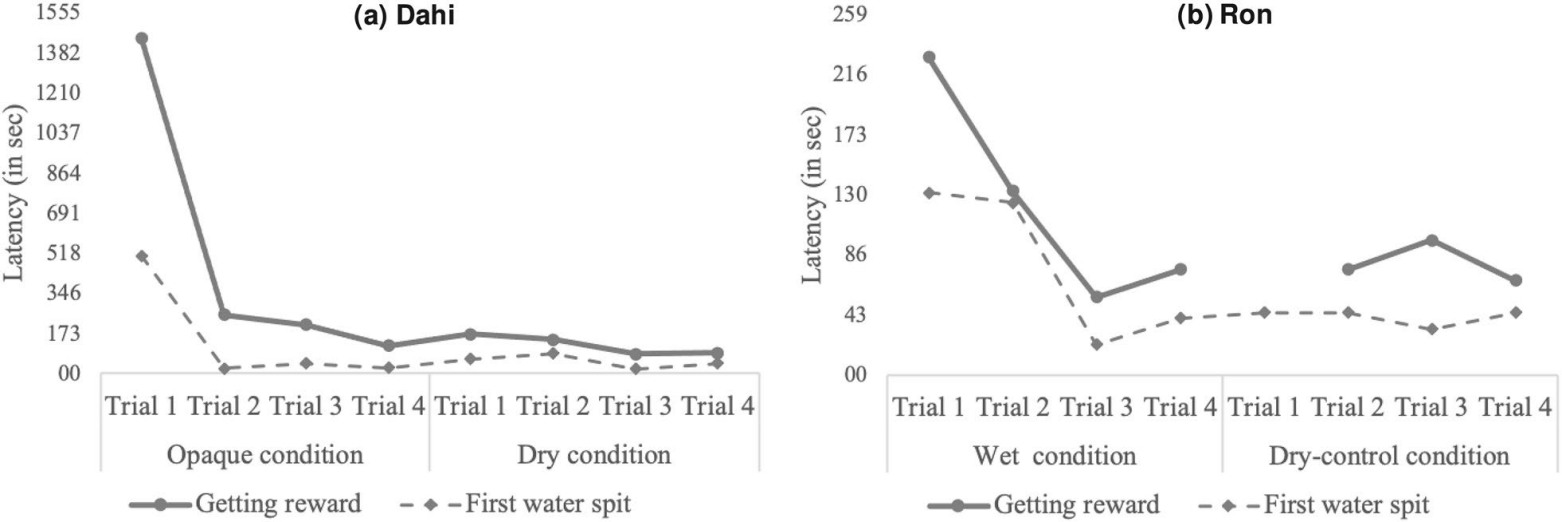
Latencies to first spit and getting the reward and frequency of (hand, foot, and mouth) manipulations for **a** Dahi across the opaque and dry trials, and **b** Ron across the wet- and dry-control trials

For Dahi, the time required to solve the problem decreased exponentially across sessions (*y* = *x*^−0.94^, adjusted *R*^*2*^ = .87, *F*_*8*_ = 53.73, *p* < .001; see Fig. 1a). It took him 1446 s to get the peanut for the first time, but only 154 s on average in the subsequent experimental sessions (latency range = 85–254 s). Dahi’s latency to first spit water into the tube also decreased sharply across sessions (*y* = *x*^−0.89^, *R*^*2*^ = .76, *F*_*8*_ = 25.94, *p* = .001, see Fig. 1a). Crucially, once the spitting behavior appeared for the first time, the frequency of tube-directed (hand/foot and mouth) actions declined dramatically, while the inter-spit latencies shortened (Supplementary file 2, Table S1). In particular, Dahi displayed a total of 50 tube-directed actions in the first trial, but only an average of 3.43 actions (frequency range = 0–7 actions) in the following experimental trials. Regarding the spitting pattern, the time lag between the first spit and the retrieval of the reward was 937 s in the first trial, but only 111 s on average (latency range = 45–232 s) in the subsequent trials. Dahi needed 4 water spits to get the reward for the first time and 3–4 water spits in the remaining experimental trials (Supplementary file 2, Table S1).

Surprisingly, Dahi only got the reward in the first dry-control condition (latency to get the reward: 149 s), but not in the remaining three dry-control trials. Yet, he spat water into the tube during the first three dry-control trials, and overall, added more water in the dry-control (mean = 1.75, total = 7 spits) compared with the table (mean = 0) and top (mean = 0.5, total = 2 spits) conditions. Throughout the course of the experiment, Dahi seemed to lose motivation to engage in the task. When arriving at the control phase, he often walked away from the tube and stayed at some distance from the experimental setup.

Ron did not add water into the tube during the opaque and dry conditions. In fact, he displayed very few actions toward the tube across the experimental conditions (Opaque: mean = 5, range = 1–9 actions; Dry: mean = 1, range = 0–4 actions). However, he managed to solve the task in the first trial of the wet-experimental condition. When presented with the dry-control condition, Ron spat water into the tube during the first trial, but was only successful from the second trial onwards (Dry control: mean = 80 s, range = 68–97 s). As for Dahi, the time needed to get the reward decreased exponentially across wet- and dry-control sessions (*y* = *x*^−0.96^, *R*^*2*^ = .91, *F*_*7*_ = 70.17, *p* < .001, see Fig. 1b): from 228 s in the first wet trial to an average of 84 s in the remaining ones (latency range = 56–132 s). Moreover, whereas in the first wet trial the time lag between Ron’s first spit and the retrieval of the reward was 97 s, in the remaining successful trials, he only needed 32 s on average (latency range = 8–64 s). Similarly, the latencies to first spit decreased exponentially over time (*y* = x^−0.94^, *R*^*2*^ = .87, *F*_*8*_ = 52.10, *p* < .001). Ron required 1 water spit to get the reward for the first time during the second wet-experimental trial, and 1–3 water spits in the remaining wet-experimental and dry-control trials (Supplementary file 2, Table S1). Regarding the tube-directed (hand/foot and mouth) manipulations, in contrast with Dahi, we did not find a clear pattern of before-after decline once Ron found the solution (Supplementary file 2, Table S1). Ron produced few actions during the opaque and dry conditions (mean = 3, range = 0–9 actions), and displayed a similar pattern during the wet-experimental and dry-control conditions (mean = 2.13, range = 0–7 actions; see Table S1). In the control conditions, Ron did not spit water into the tube and only displayed an average of 0.33 actions (range = 0–2 actions) across the top, table, and wet-control trials.

## Discussion

One of the two successful orangutans, Dahi, was able to spontaneously solve the opaque version of the FPT without any visual feedback. The solution (i.e., collecting water from the faucet and pouring it into the tube) appeared suddenly after a period of active but unsuccessful exploration, and was only used to lift the peanut out of the tube (comparison with control conditions). More importantly, in his first encounter with the opaque task, Dahi spat water into the tube a total of four times even though none of the spits produced visible changes in the peanut’s position until it finally reached the tube’s hole. In other words, his spitting behavior was not maintained by differential reinforcement (i.e., repeating those actions that bring the peanut incrementally closer to the top). This provides evidence for the first time that orangutans can solve the FPT without relying on sensorimotor learning, and it may indicate that orangutans may be capable of mentally anticipating the solution to the problem.

Despite being an intuitive problem, which does not require a complex and artificial sequence of actions, solving the FPT has proved to be highly difficult for both human and nonhuman primates, as well as other species (e.g., Bird and Emery 2009; Ebel et al. 2019a; Hanus et al. 2011). In fact, the proportion of successful human and nonhuman individuals is typically modest. Its difficulty rests, at least, on two main features. First, water is rarely used for instrumental purposes among the tested species, and its nature considerably departs from that of typical tools (liquid vs. solid); consequently, its use implies a different kind of means-end coordination. Second, the water has a strong a priori function (i.e., satiating thirst). This latter aspect has to do with functional fixedness, in the words of Shettleworth (2012), “the enemy of [mental] restructuring” (p. 218). Functional fixedness refers to a persistent reliance on past experience with a particular object in a consistent and specific way that precludes an unusual use of such object. One study suggests that this phenomenon may be responsible for so many great apes failing the FPT (Hanus et al. 2011; see also Ebel et al. 2021 for evidence of functional fixedness in other problem-solving contexts). Considering all this, our orangutan showed a sophisticated representational capacity while using water as a tool.

The only previous study of 24 naïve chimpanzees that has used the same opaque version of the FPT failed to find positive results (Ebel et al. 2019b). Although our findings should be taken very cautiously due to the limited sample size (*n* = 2), this interspecies difference is surprising as both chimpanzees and orangutans are known to show high innovation rates. Perhaps the orangutans’ unique socioecology, life history, and arboreal lifeway prepares this species for a stronger preference for exploring and using objects with the mouth (e.g., O’Malley and McGrew 2000). The mothers’ arboreal lifestyle forces their infants to use their hands to cling onto them and to use their mouth to explore the environment (Schuppli et al. 2021). These natural predispositions could generate richer and more complex sensorimotor schemes which may have favored the emergence of novel oral actions in the FPT context. Yet, considering the ensemble of FPT studies, the number of successful subjects in both chimpanzees and orangutans may have been too small to detect species differences.

At the very least, these findings indicate that success (or failure) in the FPT cannot be reduced to between-species differences. Indeed, the current study together with the previous FPT studies show substantial individual differences in performance within the species tested. A growing literature on nonhuman (and human) innovation consistently shows high rates of individual variability in innovative problem-solving that results from many sources (see Reader et al. 2016 for a review). One of the key predictors for success is the subject’s motivation to engage in the problem that is generally operationalized as both the latency to approach the task and the number of task-directed actions (Griffin and Suez 2014). Although our study design does not allow us to identify the potential contributors to the inter-individual variation observed, our results are consistent with this finding: the withdrawn subject never approached the experimental setup; and the differences in performance between the two successful orangutans mirrored their differences in active exploration of the task. The potential causes for these inter- and intra-individual differences in motivation are nevertheless unknown. Undoubtedly, the study of individual variability is a promising way to shed further light on the mechanisms underpinning innovative problem-solving across species (Kuczaj 2017).

The other successful individual, Ron, eventually got the peanut in the first trial of the original version of the FPT (wet-experimental condition) and was able to transfer this solution to the more demanding transparent version after a single trial. Also, he discriminated between experimental and control conditions, and only directed his spitting behaviors to lift the peanut out of the tube. Although we cannot conclude, in line with previous findings, that this subject innovated by means of mental combinations, the behavioral pattern displayed by Ron was similar to that of Dahi in the opaque condition, i.e., sudden appearance of the solution and exponential time decrease at getting the reward across trials, once the solution had been discovered. These data lend further support to the hypothesis that even when perceptual-motor feedback is available, great apes’ problem-solving skills seem to go beyond the scope of basic sensorimotor processes, and presumably rely on a basic understanding of the means-end relationship involved.

In conclusion, the present study shows for the first time that orangutans can potentially mentally generate the correct solution for the FPT without receiving visual information about the effect of pouring water into the tube to extract the peanut. This depends on some understanding of the means-end relationship involved. However, it is unclear whether the orangutans’ knowledge about the task is encoded as procedural or practical representations (in terms of “*what* causes the peanut reach the top of the tube”) or as abstract knowledge (in terms of “*how* the peanut reaches the top of tube”) (Seed and Mayer 2017). To further shed light on this question, more tests of the opaque version of the FPT with naïve orangutans and other great ape species (especially, chimpanzees) are needed. Those tests should systematically manipulate the causal properties involved (e.g., presenting rewards that do not float) to gain insights on the nature of great apes’ innovative problem-solving.

## Supplementary Information

Below is the link to the electronic supplementary material.

Supplementary file1 (PDF 781 KB)

Supplementary file2 (PDF 149 KB)
