# Supplementary material for: Raising the level: orangutans solve the floating peanut task without visual feedback
Source: Primates. 2021 Oct 16;63(1):33–9. doi: 10.1007/s10329-021-00952-4 (PMC8800926; doi:10.1007/s10329-021-00952-4)

Supplementary file 1

Primates

Raising the level: Orangutans solve the floating peanut task without visual feedback

Carla Sebastián-Enesco, Nerea Amezcua-Valmala, Fernando Colmenares, Natacha Mendes, and Josep Call

Correspondence concerning this article should be addressed to Carla Sebastián-Enesco, Grupo UCM de Psicobiología social, evolutiva y comparada; Departamento de Investigación y Psicología en Educación, Facultad de Psicología, Universidad Complutense de Madrid. Campus de Somosaguas 28223 Pozuelo de Alarcón, Madrid (Spain). E-mail: carla.sebastian@gmail.com

Figure 1: Drawings of (a) the tubes presented in the experimental conditions: opaque, dry, and wet-experimental; (b) the experimental setup for the opaque condition (experimenter perspective)

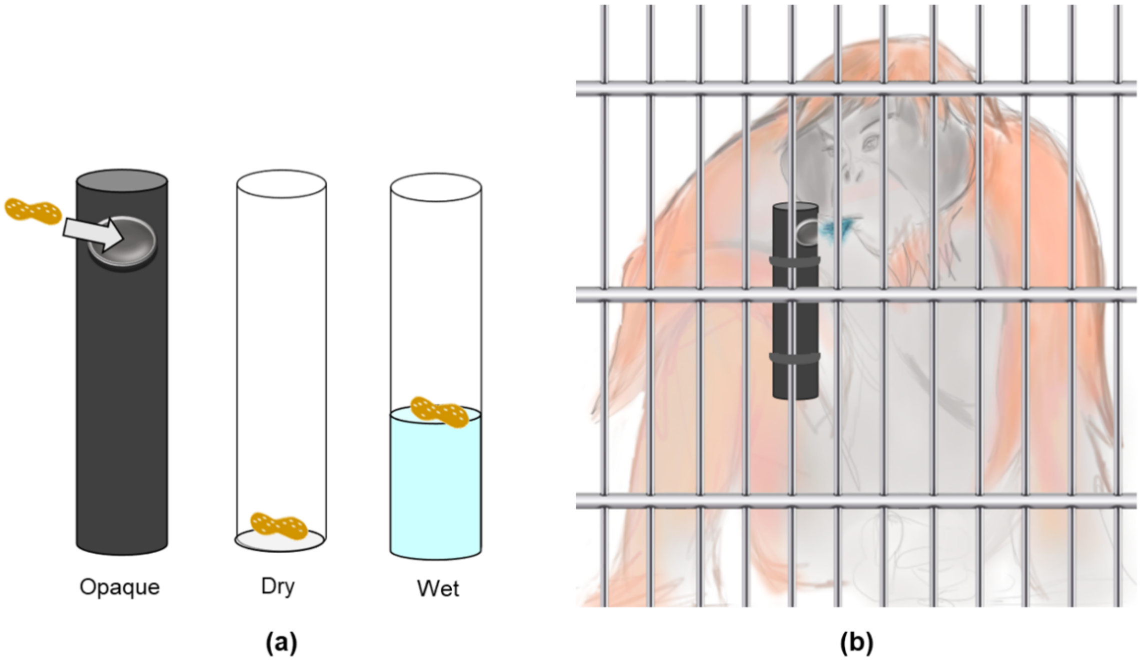

Figure 2: Schematic drawings of control conditions (subject perspective): (a) Wet-control, (b) Top, (c) Table, (d) Dry-control

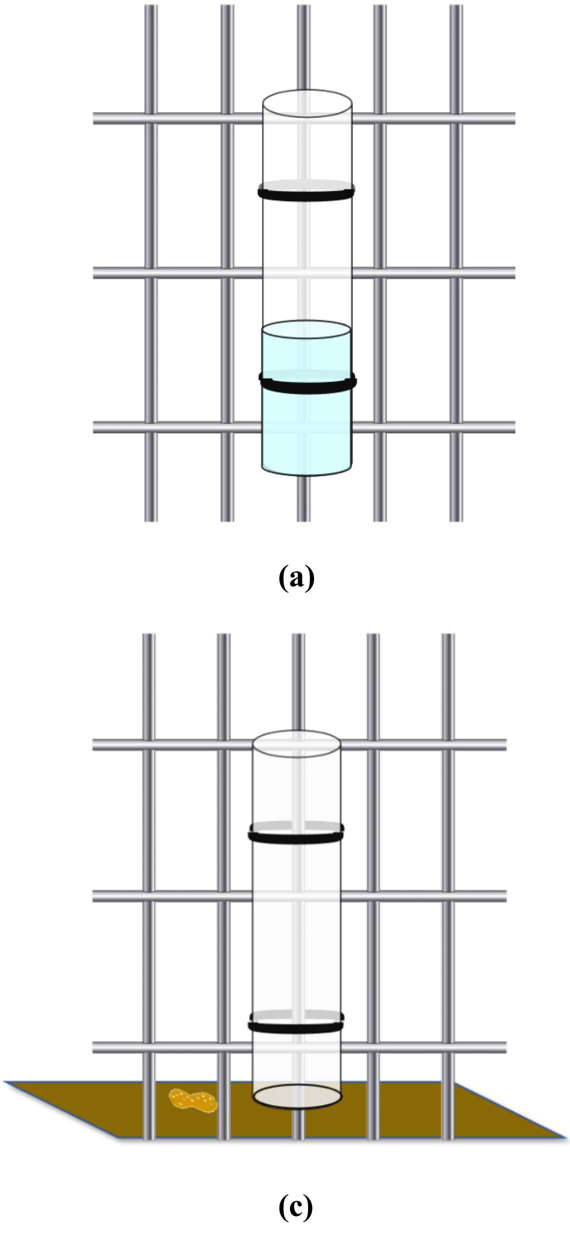

Supplement: Supplementary file 1 — Supplementary file1 (PDF 781 KB) [file 10329_2021_952_MOESM1_ESM.pdf]
