# Supplementary material for: Raising the level: orangutans solve the floating peanut task without visual feedback
Source: Primates. 2021 Oct 16;63(1):33–9. doi: 10.1007/s10329-021-00952-4 (PMC8800926; doi:10.1007/s10329-021-00952-4)
Supplement: Supplementary file 2 — Supplementary file2 (PDF 149 KB) [file 10329_2021_952_MOESM2_ESM.pdf]

Supplementary file 2

Primates

Raising the level: Orangutans solve the floating peanut task without visual feedback

Carla Sebastián-Enesco, Nerea Amezcua-Valmala, Fernando Colmenares, Natacha Mendes, and Josep Call

Correspondence concerning this article should be addressed to Carla Sebastián-Enesco, Grupo UCM de Psicobiología social, evolutiva y comparada; Departamento de Investigación y Psicología en Educación, Facultad de Psicología, Universidad Complutense de Madrid. Campus de Somosaguas 28223 Pozuelo de Alarcón, Madrid (Spain). E-mail: carla.sebastian@gmail.com

Table S1. Number of water spits and (ineffective) tube-directed actions for every trial in each condition for Dahi and Ron

Dahi												
	Opaque condition				Dry condition				Dry-control condition			
	Trial 1	Trial 2	Trial 3	Trial 4	Trial 1	Trial 2	Trial 3	Trial 4	Trial 1	Trial 2[a]	Trial 3[a]	Trial 4[a]
Water spits	4	4	4	3	3	3	4	3	3	1	1	0
Tube-directed actions[b]	50	7	7	1	6	5	1	0	4	7	5	4
Ron												
	Wet-test condition				Dry-control condition							
	Trial 1[a]	Trial 2	Trial 3	Trial 4	Trial 1[a]	Trial 2	Trial 3	Trial 4				
Water spits	2	1	2	1	2	3	3	3				
Tube-directed actions	7	2	0	0	5	0	1	2				

[a] Trials in which subjects did not get the reward.

[b] Hand/foot (pulling, lifting, banging) and mouth (biting, licking) actions.
